# Catatonia in patients with dementia

**DOI:** 10.1192/j.eurpsy.2021.1101

**Published:** 2021-08-13

**Authors:** S. Khouadja, C. Ben Taleb, R. Melki, S. Younes, L. Zarrouk

**Affiliations:** 1 Psychiatry, University Hospital Of Mahdia., Mahdia, Tunisia; 2 Psychiatry, University Hospital Of Mahdia, Mahdia, Tunisia

**Keywords:** Catatonia, psychiatry, dementia, Alzheimer

## Abstract

**Introduction:**

Catatonia has been reported with almost all types of dementia but it remains under-diagnosed.

**Objectives:**

Describe the characteristics of catatonia in patients with dementia and the efficiency of early management.

**Methods:**

We review a case of a young patient admitted in our psychiatric department for catatonia and after efficient treatment, assessment revealed a dementia.

**Results:**

A 49-year-old male treated with classic antipsychotic drug for an acute psychotic episode at age of 35 years. Three years later, the patient was admitted for behavioral disorders with delirium and confusion. The patient was treated with high-doses of antipsychotic drugs with vasodilator treatment. Currently, ten years later, he was hospitalized in a stuporous state with food refusal, sustained posture and worsening of his overall situation. At the mental assessment, the patient was motionless, mute and rigid with frozen facial expression and gaze stare. Negativity and opposition were obvious against any solicitation. Moreover, the physical examination has shown a worsening of the overall state of health, weight loss and walking difficulties. After symptomatic treatment of catatonia with benzodiazepine, the assessment revealed an aphaso-apraxo-agnotic syndrome with memory dysfunctions such as amnesia with false recognition and executive dysfunction as well as limitations in intellectual abilities. A brain scan revealed cortical and subcortical atrophy predominant in the bilateral fronto-temporo-parietal region associated with ventricular system expansion. The diagnosis of Alzheimer’s disease was made. Following atypical antipsychotic treatment combined with benzodiazepine, there was release of inhibition.

**Conclusions:**

Catatonia is a severe neuropsychiatric syndrome with an excellent prognosis if recognized and treated without delay.

